# Electromechanical Performances of Polyvinyl Chloride Gels Using (Polyvinyl Chloride-Co-Vinyl Acetate) (P(VC-VA)) Synergistic Plasticization

**DOI:** 10.3390/polym16131904

**Published:** 2024-07-03

**Authors:** Han Yan, Chang Wei, Zexing Wang, Lei Liu, Zicai Zhu, Junshi Zhang, Jihong Zhu, Weihong Zhang

**Affiliations:** 1 School of Mechanical Engineering, Northwestern Polytechnical University, Xi’an 710072, China; hanyan@mail.nwpu.edu.cn (H.Y.); hc-weichang@mail.nwpu.edu.cn (C.W.); junshi.zhang@nwpu.edu.cn (J.Z.); zhangwh@nwpu.edu.cn (W.Z.); 2 Shaanxi Key Lab of Intelligent Robots, School of Mechanical Engineering, Xi’an Jiaotong University, Xi’an 710049, China; wangzexing@stu.xjtu.edu.cn (Z.W.); zicaizhu@xjtu.edu.cn (Z.Z.); 3 Research & Development Institute of Northwestern Polytechnical University in Shenzhen, Shenzhen 518063, China

**Keywords:** PVC gel, relative dielectric constant, polyvinyl chloride-co-vinyl acetate

## Abstract

The current polyvinyl chloride (PVC) gel flexible actuators are facing challenges of high input voltage and an insufficient elastic modulus. In this study, we conducted a detailed study on the properties of PVC gel prepared by introducing the modifier polyvinyl chloride-vinyl acetate (P(VC-VA)). We compared a modified PVC gel with the traditional one in terms of the relative dielectric constant, mechanical modulus, and electromechanical actuation performance. Experimental results demonstrated that the introduction of P(VC-VA) enhanced the dielectric constant and reduced the driving electric field strength of PVC gels. The dielectric constant increased from 4.77 to 7.3. The electromechanical actuation performance increased by 150%. We employed the Gent model to fit the experimental results, and the actual experimental data aligned well with the expectations of the Gent model. The research results show that this type of plasticizing method effectively balanced the mechanical and electrical performance of PVC gels. This study summarizes the experimental results and performance analysis of PVC gels prepared using innovative plasticization methods, revealing the potential engineering applications of polymeric gels.

## 1. Introduction

In recent years, there has been a significant interest in exploring the application of electroactive polymers (EAPs) in flexible actuators and devices [1,2,3,4,5,6,7]. EAPs offer numerous advantages, including high driving strain and/or stress, high flexibility, low noise, light weight, and ease of processing and manufacturing. The most common electroactive materials can usually be divided into two types: ionic electroactive polymers (EAPs) and non-ionic EAPs. Ionic EAPs, such as conductive polymers and ionic polymer-metal composites (IPMCs), are driven by the migration and diffusion of ions and their conjugate substances. On the other hand, non-ionic EAPs, such as dielectric elastomers (DEAs) [8,9,10] and PVC gels [4], are driven by the Maxwell force generated by the electric field.

**Figure 1:**
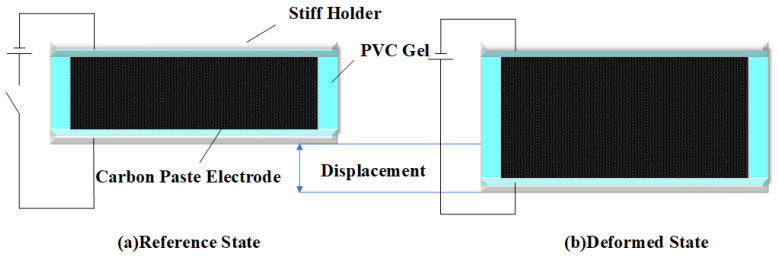
(**a**) The reference state of the PVC gel actuator when not applied electric field, and (**b**) the deformed state under an applied electric field.

DEs offer several advantages, such as large deformation (up to >380%), high stress, fast response speed, and high energy density [11]. However, achieving satisfactory performance with DEs typically requires high voltages, generally higher than 1 kV, which raises safety concerns in practical applications [12]. On the other hand, flexible actuators based on PVC gel display strain, strength, and speed comparable to human muscles at a relatively low actuation voltage (usually about 1 kV). The actuation effect of PVC gel actuators is shown in Figure 1. Their excellent stability and durability give them a high application prospect [12,13]. PVC gels have been the subject of study for several decades [5,14,15], initially being considered non-electroactive due to their low electrical conductivity, which means they cannot deform under an electric field. However, in 1999, T. Hirai et al. discovered their electroactive properties and successfully applied them to soft actuators [16]. Over the past two decades, significant research efforts have been dedicated to the development of PVC gel-based actuators and devices, generating substantial interest across interdisciplinary fields worldwide, with the ability to change in size or shape when subjected to an electrical stimulus. The square PVC gel membrane, depicted in Figure 1, serves as the reference state. On both surfaces of the PVC film, flexible electrodes are applied, resulting in a deformed state after the application of an electric field. As the understanding of PVC gels has grown, several modification techniques, such as P(VC-VA) [17], cyanoethyl cellulose(CEC) [18], ionic liquids [19], etc., have emerged. These modifications aim to reduce actuation voltage and alter the shear modulus. 

In this article, we explore the effects on PVC gel performance from introducing P(VC-VA) as a modifier for PVC. P(VC-VA) is a versatile synthetic resin widely employed in industries spanning coatings, adhesives, fibers, pharmaceuticals, and more [20]. Notable characteristics of P(VC-VA) include its high glass transition temperature (Tg), weather resistance, oil resistance, acid-base resistance, solvent resistance, and compatibility with various resins [21]. The molecular structure of P(VC-VA) includes chlorine atoms and ester groups, the introduction of these functional groups thus endows the traditional PVC gel with new properties. Previous research has explored the use of P(VC-VA) as a modifier for PVC gel to manufacture a lens, where the study only investigated the relative dielectric constant of the modified PVC gel and the performance of the lens [17]; this present study offers a more detailed investigation. Several factors can affect the deformation of PVC gel actuators. Consequently, Li conducted a study to investigate the impact of various factors, such as actuation voltage, Young’s modulus, and constitutive models on the deformation of the PVC gel [5]. To modify the characteristics of the PVC gel actuator for different scenarios, we introduced P(VC-VA). For instance, certain scenarios may necessitate a greater actuation force, improved shear modulus, or larger displacement. By testing the relative dielectric constant, shear modulus, and actuation performance of the modified polyvinyl chloride (PVC) gel, this paper introduces a method for measuring the electrical properties, shear modulus, and displacement of PVC gel. The Gent model is used to fit the experimental results and to theoretically predict the actuation performance of the PVC gel. The theoretical results are in good agreement with the experimental results. Through various tests, the optimal addition ratio of P(VC-VA) is determined, providing valuable insights for the future engineering applications of PVC gel. 

## 2. Material and Methods

### 2.1. Preparation of PVC Gels

PVC powder (Scientific Polymer Products, Inc., New York, NY, USA, Mw 275,000, CAS: 9002-86-2), P(VC-VA) powder (Scientific Polymer Products, Inc., New York, USA, Mw 115,000, CAS: 9003-22-9), tetrahydrofuran (THF) (Macklin Inc., Shanghai, China, CAS: 109-99-9, purity ≥ 99.9%), and dibutyl adipate (DBA) (Macklin Inc., Shanghai, China, CAS: 105-99-7, purity ≥ 99%) were used to fabricate the synergistic PVC gel. Different gels were produced based on the various weight ratios of PVC powder, P(VC-VA), and DBA. The PVC powder and P(VC-VA) were dissolved in THF. The PVC/P(VC-VA) blend film indicated only one glass transition temperature (Tg) of 72 °C, which indicated complete miscibility between the P(VC-VA) and PVC chains [17]. After obtaining a uniform solution, DBA was added and the mixture was stirred at 1000 rpm for 6 h at 50 °C. Then, using a doctor blade casting machine, the solution was cast onto release paper at a temperature set to 40 °C. After casting, the mixture was left to stand for 30 min, removed from the release paper, and finally, after standing at room temperature for 72 h, there was no THF residue remaining in the gel.

The experimental group PVCG (PVC/DBA) kept the PVC content constant (10 g), while the DBA content gradually increased (30 g, 40 g, 60 g, and 80 g). The purpose of this group was to observe the impact of increasing DBA content on the properties of PVC gel, as shown in Table 1. 

The experimental group PVAG (PVC/P(VC-VA)/DBA) maintained the contents of PVC and P(VC-VA) constant (5 g each), with the ratio of PVC to P(VC-VA) maintained at 1:1. Similar to the first group, the DBA content gradually increased (30 g, 40 g, 60 g, and 80 g). The aim of this group was to compare the performance differences between traditional PVC gel and PVC gel modified with P(VC-VA) at the same DBA content levels, as shown in Table 2. 

The experimental group PPG (PVC/P(VC-VA)/DBA) kept the DBA content constant (80 g), while PVC was gradually replaced entirely by P(VC-VA) (the PVC content decreased from 20 g to 0 g and the P(VC-VA) content increased from 0 g to 20 g). The purpose of this group was to study the performance changes in the gel as PVC is replaced by P(VC-VA), as shown in Table 3.

### 2.2. Relative Dielectric Constant Measurements

**Figure 2:**
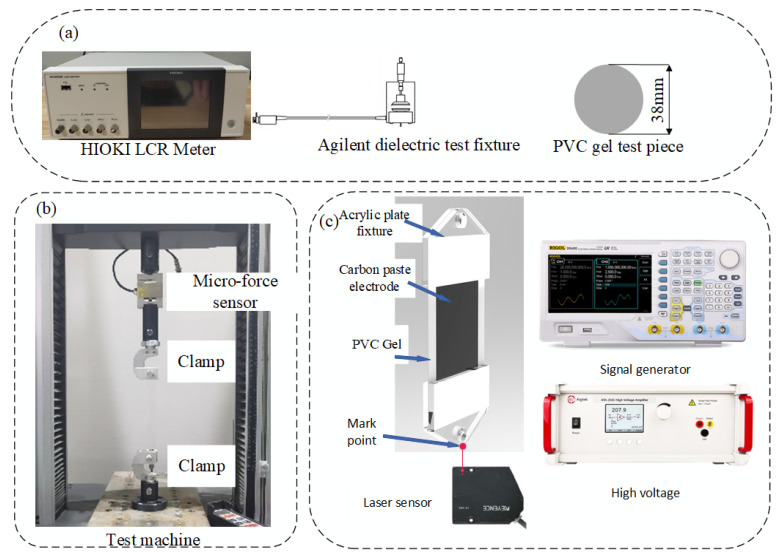
(**a**) The relative dielectric constant of the gel was tested using an LCR meter and a dielectric test fixture. (**b**) The stress–strain relationship of the gel was tested using a tensile testing machine. (**c**) A sheet-type actuator was constructed to measure displacement, employing a laser displacement sensor.

To measure the relative dielectric constant, the PVC gel samples were cut into a circular shape with a diameter of 38 mm. The tests were conducted using the HIOKI LCR Meter (IM3536, HIOKI E.E. Corporation, Nagano, Japan) with an Agilent dielectric test fixture (16451B, Agilent Technologies Co., Ltd., Santa Clara, CA, USA) under a low electric field condition of 1 V/mm, as shown in Figure 2a. We performed relative dielectric constant measurements across a frequency range of 4–10^6^ Hz. To minimize the effect of temperature on deformation [22], all tests were carried out at the same temperature. 

### 2.3. Mechanical Tension Measurements

The tensile stress–strain of samples was measured using an electronic universal testing machine (Senstest Co., Ltd., Shenzhen, China). The samples, with dimensions of 25 mm × 100 mm, were clamped and extended uniaxially at a rate of 5 mm/min until failure occurred at the sample’s midpoint, as shown in Figure 2b. Each sample was tested three times. The shear modulus was determined by the initial slope of the stress–strain curve.

### 2.4. Electromechanical Displacement Measurements

To investigate the effect of P(VC-VA) on the actuation performance, we conducted an electromechanical displacement measurement. We used absolute electric field strength to determine the required voltage. Due to problems with the molding process, the thickness of our PVC gel samples was 200 μm ± 20 μm. Based on this, we calculated and applied a voltage range of 0 V to 3000 V. Acrylic plates were utilized to clamp the upper and lower sides of the PVC gel, while carbon grease electrodes were brushed to both sides of the PVC gel. A 1 mm gap was left at the edges to prevent electrode short-circuiting. The signal generator used DG4000 (RIGOL TECHNOLOGIES Co., Ltd., Suzhou, China) and the voltage amplifier used ATA-2161 (Xi’an Antai Electronic Technology Co., Ltd., Xi’an, China). Experimental data were obtained using a laser displacement sensor Keyence LK-G80 (KEYENCE CORPORATION., Shanghai, China) to measure the marker point on the lower acrylic plate, as shown in Figure 2c.

## 3. Results and Discussion

### 3.1. Relative Dielectric Constant Measurements

The relative dielectric constant of the PVC gel is very high at low frequencies. As the frequency increases, the relative dielectric constant decreases and finally stabilizes. This is because the drastic change in the relative dielectric constant indicates a strong interaction between PVC and DBA. Under the influence of an electric field, DBA molecules become charged and polarized. These polarized molecules are able to track the changes in the polarity of the electric field at low frequencies, thereby facilitating the dipole rotation of the PVC chain segments. Moreover, the simultaneous polarization of the DBA and PVC chains enhances the overall polarization, leading to a significant increase in the relative dielectric constant of the gel. Notably, the relative dielectric constant of the PVC gel continuously decreases with the increase in frequency until 100 Hz and remains constant between 100 and 1000 Hz [23].

#### 3.1.1. Effects of DBA and P(VC-VA) on Relative Dielectric Constant

**Figure 3:**
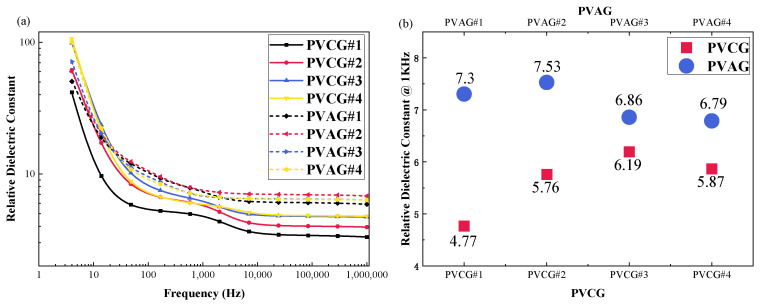
(**a**) The frequency range is $4–10^{6}\;Hz$, and the test results of the relative dielectric constant for the experimental groups PVCG (PVC/DBA) and PVAG (PVC/P(VC-AC)/DBA) are shown. (**b**) The relative dielectric constant for both experimental groups at the frequency of 1000 Hz.

The relative dielectric constant test results for the PVCG and PVAG groups are shown in Figure 3a, which demonstrates that the incorporation of P(VC-VA) leads to an increase in the relative dielectric constant of the corresponding PVAG. Figure 3b provides a more direct observation of the increase in all relative dielectric constants at a frequency of 1 kHz. The experiments with PVCG and PVAG confirm that adding P(VC-VA) to PVC gels with different PVC:DBA ratios can significantly enhance their relative dielectric constant, with PVAG#1 showing a 53% increase over PVCG#1. M. Ali’s research indicates that mixing DBA into PVC gels will reduce the relative dielectric constant when the proportion exceeds a certain threshold [23]. This study also shows that continuously adding DBA to PVC gels containing P(VC-VA) in an approximate ratio of 1:1:8 will similarly result in a decrease in the relative dielectric constant. 

#### 3.1.2. Effect of P(VC-VA) on Relative Dielectric Constant

**Figure 4:**
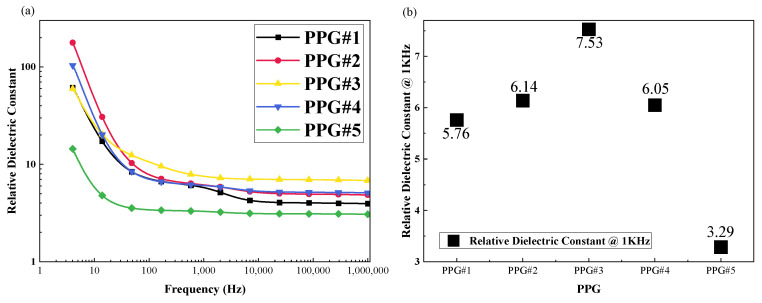
(**a**) The frequency range is $4–10^{6}\;Hz$, and the test results of the relative dielectric constant for the experimental groups PPG(PVC/P(VC-AC)/DBA) are shown. (**b**) The relative dielectric constant for the PPG experimental group at the frequency of 1000 Hz.

The results of the PPG group are shown in Figure 4a. The incorporation of P(VC-VA) from PPG#1 results in an increased relative dielectric constant for the PVC gel, reaching a peak at PPG #3. As more P(VC-VA) replaces PVC, there is a gradual decline in the relative dielectric constant. Figure 4b offers a more visual representation, indicating that PPG #3 possesses the highest relative dielectric constant at a frequency of 1 kHz, with a value of 7.53. The experiments conducted within the PPG group demonstrate that an excessive introduction of P(VC-VA) leads to a decrease in the relative dielectric constant, particularly when comparing PPG #4 and PPG #5, where a significant reduction is observed, with the relative dielectric constant dropping from 6.05 to 3.29. This indicates that P(VC-VA) cannot be used alone in the preparation of PVC gels. Although PPG#5 itself does not contain PVC, we use the term “PVC gels” to maintain consistency in terminology across the entire PPG series.

### 3.2. Mechanical Tension Measurements

The mechanical deformation behavior of hyperelastic materials is commonly described using strain energy density functions. In the mechanical research of DE, several commonly used hyperelastic models exist to describe the relationship between deformation and energy, including the Mooney–Rivlin model [24], the Yeoh model [25], the Ogden model [26], and the Gent model [27].

The Gent model considers the stretching limit of molecular chains and can explain the stress–stiffening phenomenon that occurs under large deformation. In this article, the Gent model is used as the fitting model.

#### 3.2.1. The Joint Effect of DBA and P(VC-VA) on the Mechanical Modulus

**Figure 5:**
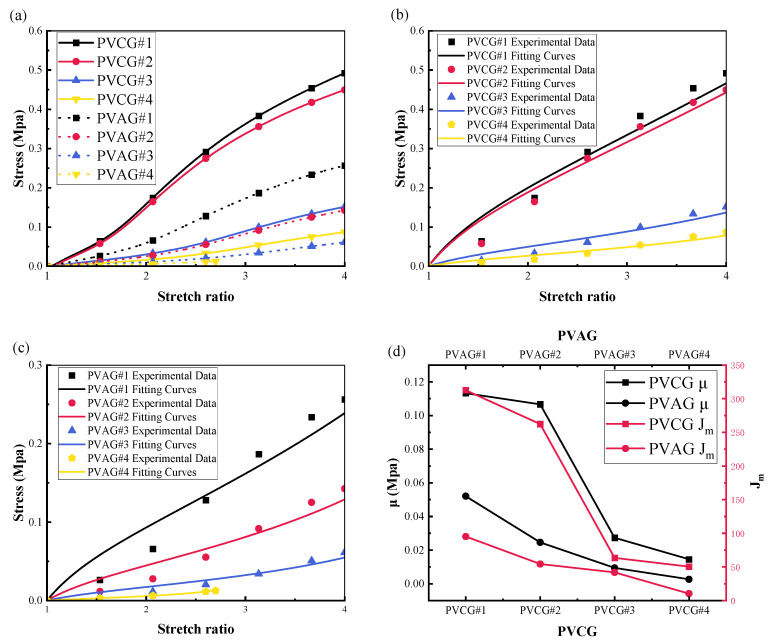
A comparative analysis is presented for the PVCG (PVC/DBA) and PVAG (PVC/P(VC-AC)/DBA) experimental groups, focusing on the following aspects: (**a**) the stress–strain relationship of both the PVCG and PVAG groups, (**b**) the comparison between the experimental data and the fitted data within the PVCG group, (**c**) the comparison between the experimental data and the fitted data within the PVAG group, and (**d**) the variable relationship between μ and $J_{m}$ pertaining to the changes in DBA and P(VC-VA) after applying the Gent model for data fitting in both the PVCG and PVAG groups.

The experimental results of the stress–strain relationship are presented in Figure 5a, which shows the results for both the PVCG and PVAG groups. The experimental data reveal that the mechanical tension in the PVAG group is lower than that in the PVCG group, indicating that the introduction of P(VC-VA) reduces the elastic modulus of the PVC gel, making it softer. In the PVCG group, a decrease in the mass fraction of DBA corresponds to an increase in the elastic modulus, in the order of PVCG#1 > PVCG#2 > PVCG#3 > PVCG#4. Similarly, in the PVAG group, the smaller the mass fraction of DBA, the higher the elastic modulus. PVAG#4 fractured at approximately 180% strain, indicating that it is not suitable for applications that require a high output force. Like other hyperelastic models, an elastic strain energy function is used to describe the model. Specifically, the elastic strain energy function associated with the Gent model is expressed as follows:

(1) $$W_{Gent}=-\frac{\mu J_{m}}{2}\ln\left(1-\frac{I_{1}-3}{J_{m}}\right)$$

In Equation (1), $I_{1}$ represents the first invariant of the strain tensor. μ is the shear modulus of the superelastic material. $J_{m}$ is the maximum deformation limit of the molecular chain of the superelastic material. We consider polyvinyl chloride gel as an incompressible material with a Poisson’s ratio of 0.5 under incompressible conditions [18]. By utilizing Equation (1), we can acquire the desired outcome.

(2) $$W_{Gent}=-\frac{\mu J_{m}}{2}\ln\left(1-\frac{{\lambda_{1}}^{2}+{\lambda_{2}}^{2}+{\lambda_{3}}^{2}-3}{J_{m}}\right)$$

Equation (2) defines $I_{1}={\lambda_{1}}^{2}+{\lambda_{2}}^{2}{{+\lambda }_{3}}^{2}$ as the sum of squares of $\lambda_{i}\left(i=1,2,3\right)$, which correspond to the stretching ratios of the thin film along its three principal directions. The values of $\lambda_{i}=L_{i}/L_{i0}$ are not dependent on the material’s dimensions, where $L_{i}$ and $L_{i0}$ represent the stretched length and initial length, respectively, of the thin film in the i-direction. Considering the incompressibility of DE materials, $\lambda_{1}\lambda_{2}\lambda_{3}=1$, it is required that the stretching ratios along the three directions of the thin film adhere to the following relationship during uniaxial stretching:

(3) $$\lambda_{2}=\lambda_{3}=\frac{1}{\sqrt{\lambda_{1}}}$$

Based on the Gent model, we can determine the Cauchy stress (true stress) magnitude of in the direction of stretching 1:

(4) $$\sigma =\frac{\partial W_{Gent}}{\partial \lambda_{1}}=-\frac{\mu }{2}\ln\left(\frac{{\lambda_{1}}^{2}-2{\lambda_{1}}^{-2}}{1-\frac{\left(2{\lambda_{1}}^{-1}+{\lambda_{1}}^{2}-3\right)}{J_{m}}}\right)$$

By using Formula (4) to fit the experimental results, we can obtain the values of μ and $J_{m}$ under different contents of PVC, P(VC-VA), and DBA. The comparison between the fitting results and the experimental results of the PVCG group is shown in Figure 5b, and the comparison for the PVAG group is also shown in Figure 5c. It can be observed that the Gent model fits the PVC gel well. The relationship between μ and $J_{m}$ and the experimental groups of PVCG and PVAG is shown in Figure 5d. It can be seen that with the introduction of P (VC-VA), μ and $J_{m}$ both decrease, which shows that the shear modulus of PVC gel and the maximum deformation limit of the molecular chain decrease. This is caused by the VA grafted on PVC destroying the strong secondary bonds between PVC chains, thereby reducing the intermolecular force between PVC chains [17].

#### 3.2.2. Effect of P(VC-VA) on Mechanical Modulus

**Figure 6:**
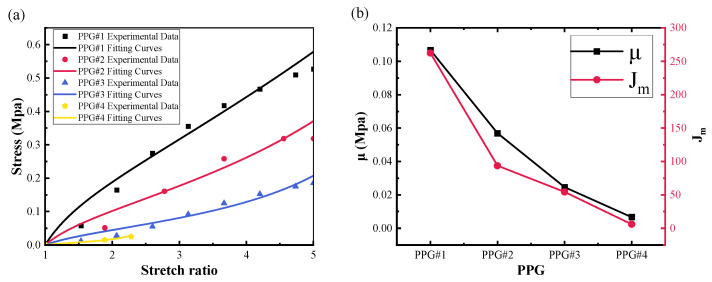
A comparative analysis is performed on the experimental data and fitted data from the PPG(PVC/P(VC-AC)/DBA) groups, focusing on the following aspects: (**a**) the correlation between the experimental and fitted data within the third group, and (**b**) the variable relationship between μ and $J_{m}$ pertaining to the changes in DBA and P(VC-VA) after applying the Gent model for data fitting in the third group.

Figure 6a shows the comparison between the PPG group model and the fitting results. It can be observed that as the mass fraction of P(VC-VA) increases, the elastic modulus of the PVC gel gradually decreases, indicating that the PVC gel becomes softer. This result suggests that the combination of PVC gel with P(VC-VA) may not be suitable for applications requiring high output force. Notably, PPG#4 did not achieve a strain of 200%, and PPG#5 could not undergo the tensile test. As we motioned earlier in Section 3.1.2, these observations further confirm that P(VC-VA) cannot completely replace PVC in the preparation of PVC gels. Figure 6b delineates the relationship between μ and $J_{m}$ in relation to the PPG groups.

### 3.3. Voltage-Induced Actuation Performance Measurements

The actuation experiments of PVC gel are primarily conducted to verify the relationship between displacement and electric field strength and to detect the electric field strength at which PVC gel fails due to dielectric breakdown. These parameters are of significant importance for various future applications. Initial experiments tested the relative permittivity of PVC gel, and it was observed that an increase in permittivity directly leads to a reduction in the actuation voltage. Therefore, these tests indicate that the introduction of P(VC-VA) could potentially lower the actuation voltage.

Under the influence of the electric field, the Maxwell stress relates as follows:

(5) $$\sigma_{Maxwell}=\varepsilon_{0}\varepsilon_{r}E^{2}$$

Thus, the deformation of the PVC gel is equivalent to biaxial deformation.

(6) $$\lambda_{1}=\lambda_{2}=\frac{1}{\sqrt{\lambda_{3}}}$$

Upon incorporating Equation (5) into the Gent model, the resultant electromechanical coupling relationships within the material can be ascertained as follows:

(7) $$\sigma =\lambda_{1}\frac{\partial W_{Gent}}{\partial \lambda_{1}}=\mu \left(\frac{\lambda_{1}^{2}-\lambda_{1}^{-4}}{1-\frac{\left(2\lambda_{1}^{2}+\lambda_{1}^{-4}-3\right)}{J_{m}}}\right)-\varepsilon_{0}\varepsilon_{r}E^{2}$$

During the actuation tests, where an external force load is absent (*σ* = 0) and only an electrical load is present, the correlation between the electric field and deformation can be determined as follows:

(8) $$\varepsilon_{0}\varepsilon_{r}E^{2}=\lambda_{1}\frac{\partial W_{Gent}}{\partial \lambda_{1}}$$

The detailed expression is as follows:

(9) $$E=\sqrt{\frac{\left(\frac{\mu \left(\lambda^{2}-\lambda^{-4}\right)}{1-\frac{\left(2\lambda^{2}+\lambda^{-4}-3\right)}{J_{m}}}\right)}{\varepsilon_{0}\varepsilon_{r}}}$$

#### 3.3.1. The Joint Effect of DBA and P(VC-VA) on Actuation Performance

**Figure 7:**
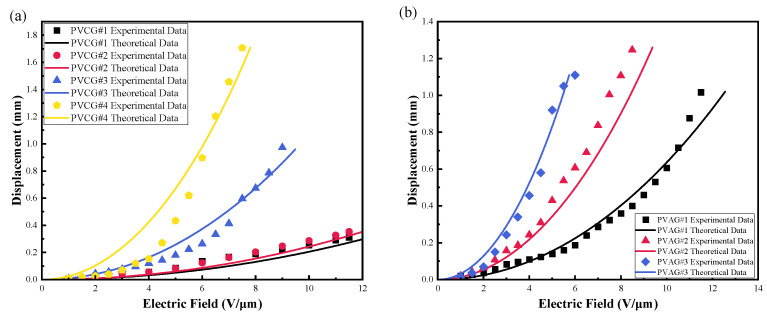
The experimental results and theoretical calculation data of the actuation performance of the (**a**) PVCG (PVC/DBA) and (**b**) PVAG (PVC/P(VC-AC)/DBA) groups under the electric field.

Figure 7a shows the relationship between the experimental data and theoretical data for the PVCG group’s actuation experiment. Figure 7b shows the relationship between the experimental data and theoretical data for the PVAG group’s actuation experiment. As the mass fraction of DBA increases, the displacement increases under the same electric field strength, but the breakdown field strength decreases, and the breakdown field strengths of PVCG #1, PVCG #2, PVCG #3, and PVCG #4 are 11.5 V/μm, 11.5 V/μm, 9.5 V/μm, and 7.5 V/μm, respectively. The breakdown field strengths of PVAG #1, PVAG #2, and PVAG #3 are 12.5 V/μm, 9.5 V/μm, and 5.5 V/μm, respectively. This is because when the displacement of the PVC gel increases, the thickness decreases, and the actual electric field strength becomes greater than the absolute electric field strength. The comparison between Figure 7a,b clearly shows that the introduction of P(VC-VA) increased the relative dielectric constant and reduced the actuation electric field strength of the PVC gel. For the comparison groups PVCG#1 and PVAG#1 at an electric field strength of 10 V/μm, the displacement of PVAG#1 increased by 150% compared to PVCG#1, and there were relative increases for the other comparison groups as well. These findings are consistent with our observations of the increase in the relative dielectric constant, which reduced the actuation electric field strength of the PVC gel. PVAG#4 deserves special mention because the material has a relatively low elastic modulus and is softer, which results in a certain degree of initial stretching during the experiment due to gravity. This stretching caused by gravity affects the calculation of the field strength, so no actuation performance experiments were conducted for PVAG#4 and PVCG#5.

#### 3.3.2. Effect of P(VC-VA) on Actuation Performance

**Figure 8:**
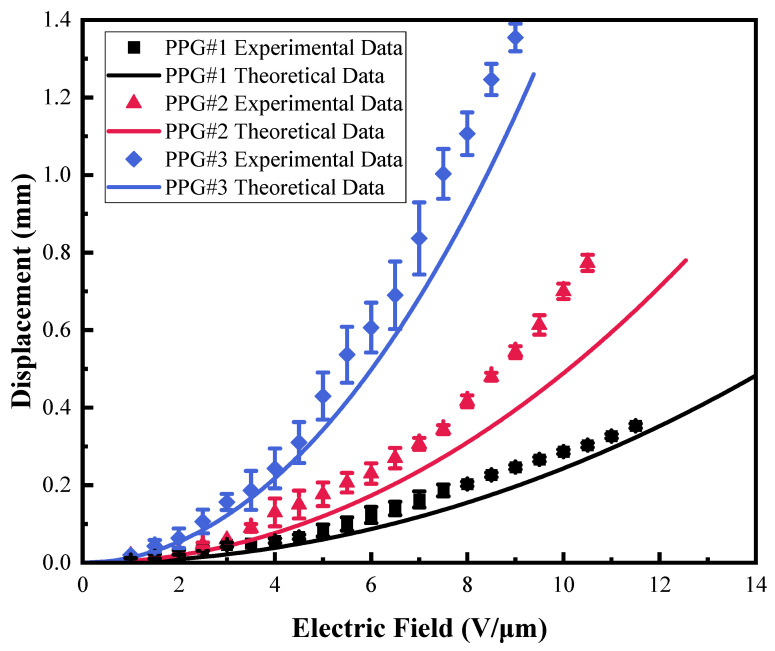
The experimental results and theoretical calculation data of the displacement actuation performance of the PPG(PVC/P(VC-AC)/DBA) test group under the electric field.

The experimental results and theoretical calculations of the PPG group’s actuation performance test are shown in Figure 8. It can be seen from the figure that when the displacement is 0.3 mm, the electric field strengths of PPG#1, PPG #2, and PPG #3 are 11.5 V/μm, 7.5 V/μm, and 4.5 V/μm, respectively. At this same displacement, the PVC gel with added P(VC-VA) shows a lower actuation electric field strength. This aligns with our relative dielectric constant test results, confirming that as long as the mass fraction of P(VC-VA) does not exceed 50%, the larger the mass fraction, the lower the actuation electric field strength of the PVC Gel. However, P(VC-VA) should not be added excessively, nor should it be used alone to prepare the gel. Furthermore, the introduction of P(VC-VA) also causes a decrease in the breakdown field strength of the PVC gel, with the breakdown field strengths of PPG #1, PPG #2, and PPG #3 being 11.5 V/μm, 10.5 V/μm, and 8 V/μm, respectively. Among them, PPG#1 has the worst actuation effect, while PPG#3 has the best.

## 4. Conclusions

This study investigates the use of P(VC-VA) as a synergistic plasticizer in the study of PVC gels. The purpose is to address the issues of high input voltage requirements and a low elastic modulus in existing PVC gel flexible actuators. In addition, the evaluation technology of the electrical properties, the elastic modulus, and the displacement of PVC gel is also introduced, and the Gent model is used to theoretically estimate the elastic modulus and driving performance of PVC gel, from which we reached the following conclusions:

1. Conclusions can be drawn from the comparison between the PVCG (PVC/DBA) experimental group and the PVAG (PVC/P(VC-AC)/DBA) experimental group. After the introduction of P(VC-VA), the dielectric constant of the synergistically plasticized PVC gel is improved. The dielectric constant of PVCG#1 is increased from 4.77 to 7.3 of PVAG#1. This improvement is beneficial to improving the electric drive performance of PVC gel.
2. After the introduction of P(VC-VA), the elastic modulus of synergistically plasticized PVC gel has a certain change. The electromechanical actuation performance increases by 150%. We used the Gent model to fit the experimental results, and the experimental results are in good agreement with the theoretical data.
3. The driving electric field intensity of the synergistically plasticized PVC gel is reduced after the introduction of P(VC-VA). We also found that P(VC-VA) cannot completely replace PVC in PVC gel synthesis, the mass fraction of P(VCVA) does not exceed 75%, and PVC gel with a P(VC-VA) content of 75% can be prepared, but it is too soft and is difficult to prepare the actuator, as evidenced by the inability to construct an actuator with PPG#5.

There are many ways to modify PVC gel besides introducing P (VC-VA), but this method is relatively simple to prepare, and can effectively increase the dielectric constant and reduce the driving electric field intensity, which is very important for the future use of PVC gel. P (VC-VA) is of great significance in preparing different actuators and improving performance.

## Figures and Tables

**Table 1:** The detailed ratios of the first experimental group ensured that the PVC content remained constant while gradually increasing the DBA content at 30 g, 40 g, 60 g, and 80 g, respectively.

	PVCG#1	PVCG#2	PVCG#3	PVCG#4
PVC	10 g	10 g	10 g	10 g
DBA	30 g	40 g	60 g	80 g

**Table 2:** The detailed ratios of the second experimental group ensured that the content of PVC and P(VC-VA) remained constant, and the ratio of PVC to P(VC-VA) was maintained at 1:1. The content of DBA gradually increased at increments of 30 g, 40 g, 60 g, and 80 g.

	PVAG#1	PVAG#2	PVAG#3	PVAG#4
PVC	5 g	5 g	5 g	5 g
P(VC-VA)	5 g	5 g	5 g	5 g
DBA	30 g	40 g	60 g	80 g

**Table 3:** The detailed ratio of the third group of experiments ensured that the content of DBA was 80 g, and PVC was gradually replaced completely by P (VC-VA) with an increment of 5 g per group.

	PPG#1	PPG#2	PPG#3	PPG#4	PPG#5
PVC	20 g	15 g	10 g	5 g	0 g
P(VC-VA)	0 g	5 g	10 g	15 g	20 g
DBA	80 g	80 g	80 g	80 g	80 g

## Data Availability

The original contributions presented in the study are included in the article, further inquiries can be directed to the corresponding author/s.
